# Benzo[a]pyrene-Induced Developmental Toxicity in *Caenorhabditis elegans*: Potential Involvement of Insulin/IGF Signaling and Collagen Gene Dysregulation

**DOI:** 10.3390/toxics13050384

**Published:** 2025-05-09

**Authors:** Jinjin Zhou, Yage Shi, Yanfeng Zhou, Yang Ge

**Affiliations:** School of Public Health, Shanghai Jiao Tong University School of Medicine, Shanghai 200025, China; zjj19980920@sjtu.edu.cn (J.Z.); xiao-gua@sjtu.edu.cn (Y.S.); yfzhou@shsmu.edu.cn (Y.Z.)

**Keywords:** benz[a]pyrene, *Caenorhabditis elegans*, developmental toxicity, cuticle collagen

## Abstract

Benzo[a]pyrene (B[a]P) is a widespread and persistent organic pollutant that poses serious threats to human health. Although its carcinogenic properties have been extensively studied, its developmental toxicity and underlying mechanisms remain poorly understood. In this study, we employed *Caenorhabditis elegans* (*C. elegans*) as a model organism to investigate the effects of B[a]P exposure during early developmental stages. To comprehensively assess B[a]P-induced developmental toxicity, we employed high-throughput sequencing along with transgenic and mutant *C. elegans* strains. Exposure to B[a]P at concentrations exceeding 1 mg/L significantly reduced larval body size, decreased the number of adult worms, and delayed larval-to-adult development. Furthermore, we analyzed the expression of genes involved in cuticle collagen synthesis and key components of the insulin/insulin-like growth factor signaling (IIS) pathway, including daf-2 and daf-16. These findings suggest that B[a]P-induced developmental toxicity may be associated with dysregulation of the IIS pathway. Specifically, B[a]P appears to influence the activity of the downstream transcription factor daf-16, thereby altering the expression of collagen-related genes. This disruption in collagen synthesis may contribute to delayed larval development and impaired maturation. Our study provides new insights into the environmental hazards associated with B[a]P exposure and reveals a potential mechanism underlying its developmental toxicity. Moreover, our findings highlight the critical role of collagen gene regulation during early developmental stages. These genes may serve as potential biomarkers for environmental toxicant exposure, particularly in vulnerable populations such as children undergoing critical periods of development.

## 1. Introduction

Polycyclic aromatic hydrocarbons (PAHs) are organic pollutants generated from the incomplete combustion and pyrolysis of organic materials such as biomass, petroleum, and coal. Over the past decade, numerous studies have characterized their sources, analytical characterization, bioavailability, and associated health effects [1,2,3]. PAHs exhibit high volatility, extensive environmental distribution, and strong lipophilicity. The high volatility of PAHs allows them to be widely dispersed and transported over long distances from the original source. This leads to their presence and accumulation in various environmental matrices (including water, air, sediment, and food), thus posing potential health risks [4]. Due to their lipophilic nature, PAHs are also considered food contaminants, frequently detected in lipid-rich matrices such as edible fats and oils [5]. Human exposure to PAHs primarily occurs through ingestion, inhalation, and dermal absorption [6,7]. Several studies have reported the presence of PAH compounds in human urine, blood, and breast milk [8,9]. Previous reports have demonstrated that PAH exposure may lead to a variety of adverse health outcomes, including oxidative stress, inflammation, and impaired fetal development [10,11,12].

Benzo[a]pyrene (B[a]P), a representative compound of PAHs, is widely used as a model pollutant for evaluating the toxicological effects of persistent organic pollutants (POPs). According to reports from the Canadian Great Lakes region, B[a]P concentrations in uncontaminated surface waters generally range from 0.03 to 0.7 ng/L (Health Canada, 2020). In contrast, B[a]P levels are substantially higher in industrially impacted areas, such as near coking plants or petrochemical facilities. Studies have shown that B[a]P concentrations in such contaminated waters or wastewater can reach 1–5 mg/L, particularly following episodic discharges or sediment resuspension events (ATSDR, 2020). Drinking water guidelines reflect its toxic potential, with regulatory limits ranging from 0.01 µg/L in the European Union to 0.7 µg/L established by the World Health Organization. Based on this environmental data, the concentration range used in the present study (1–4 mg/L) was selected to simulate worst-case exposure scenarios in pollution hotspots, thereby providing ecotoxicological insights relevant to high-risk environments. Ecotoxicological studies have shown that exposure to B[a]P causes developmental toxicity in both animals and humans, including embryonic developmental defects. For instance, chronic maternal intake of B[a]P during pregnancy has been linked to reduced birth weight in offspring [13]. In rats, exposure to environmentally relevant doses of B[a]P (0, 0.1, 1.0, or 10 μg/kg) during organogenesis has been shown to adversely affect fetal development [14]. While numerous studies have confirmed that high concentrations of B[a]P elicit developmental toxicity across species, the underlying mechanisms of B[a]P-induced developmental toxicity at environmentally relevant concentrations remain unclear. Given the increasing prevalence of environmental exposure, further investigation is warranted to elucidate the effects and mechanisms of B[a]P-induced developmental toxicity at environmentally relevant concentrations.

From the embryonic stage onwards, the extracellular matrix (ECM) plays a pivotal role in organismal development, characterized by tightly regulated, rapid, and continuous ECM remodeling [15]. The primary components of the ECM include collagen, laminins, proteoglycans, and glycosaminoglycans, which provide extracellular developmental cues and structural support during embryogenesis [16]. In humans, ECM abnormalities are linked to developmental disorders such as Marfan syndrome, Ehlers–Danlos syndrome, and Bethlem myopathy [17]. Collagen not only serves as a key structural element but also functions as an essential nutrient source and a critical modulator of growth [18]. During fetal and postnatal development, collagen fibers undergo dynamic changes in size and abundance, eventually becoming the most predominant structural component of adult skeletal tissue [19]. As the principal matrix constituent of cartilage, collagen is essential for proper cartilage and skeletal development; abnormalities in collagen content can impair these processes. Mutations or deficiencies in collagen are associated with conditions such as osteoporosis and skeletal dysplasia [19]. Epidemiological studies suggest that human exposure to PAHs through smoking or cooking activities contributes to skeletal disorders such as osteoporosis, characterized by reduced bone mineral density and increased fracture risk [20,21].

*Caenorhabditis elegans (C. elegans),* a well-established invertebrate model organism, is widely used in ecotoxicology and developmental toxicity research due to its short life cycle, well-defined developmental stages, and ease of continuous observation. Furthermore, it has many transgenic strains with fluorescent markers and mutant strains, and its genome shares up to 80% homology with humans, making it an excellent model for ecotoxicological and developmental toxicity research [22,23,24,25,26]. For instance, early-life exposure of *C. elegans* to the pesticide CMIT/MIT has been shown to induce metabolic disorders via the O-GlcNAc transferase pathway [24].

In this study, we employed *C. elegans* to investigate the developmental toxicity and potential mechanisms of B[a]P at environmentally relevant concentrations, aiming to predict the impact of B[a]P on early developmental processes in environmental organisms. Our results suggest that B[a]P may exert adverse effects on *C. elegans* development by downregulating collagen synthesis genes such as dpy-17 through modulation of the insulin-like signaling pathway, leading to delayed larval development and a reduced proportion of adult worms.

## 2. Materials and Methods

### 2.1. C. elegans Strains

Wild-type N2, mutant dpy-17 (e164), and TP12: Kals [COL-19: GFP] strains were obtained from Soochow University. Nematodes were cultured on nematode growth medium (NGM) plates inoculated with E. coli OP50 at a constant 20 °C in a biochemical incubator. To obtain synchronized nematodes, eggs were extracted from gravid hermaphrodites using a bleaching solution and incubated overnight at 20 °C to produce synchronized L1 larvae for subsequent experiments.

### 2.2. Preparation and Exposure of B[a]P Solution

B[a]P (from Macklin, Shanghai, China) was dissolved in DMSO to prepare a 1 g/L stock solution. Based on environmental concentrations [27,28,29], we selected 1 mg/L, 2 mg/L, and 4 mg/L as the relevant concentrations, using S Medium as the control. At the start of the experiment, 8 mL of S Medium was mixed with a concentrated E coli OP50 bacterial suspension and evenly distributed into a six-well plate, followed by incubation. B[a]P was added to each well at final concentrations of 1 mg/L, 2 mg/L, and 4 mg/L, respectively, while the control group contained only the medium without B[a]P. Synchronized L1 larvae were centrifuged at 3000× *g* rpm for 5 min. Subsequently, 30 μL of the larval suspension was introduced into each well and cultured for 36 h. The S Medium comprises 1 L of S Basal, 10 mL of 1 M potassium citrate, 3 mL of 1 M CaCl_2_, and 10 mL of a trace metal solution. The S Basal solution contains 0.1 M sodium chloride, 0.007 M K_2_HPO_4_, 0.034 M KH_2_PO_4_, and 0.013 M cholesterol. The trace metal mix includes 0.9976 M potassium citrate trihydrate and 0.00499 M EDTA Na_2_·2H_2_O. *E. coli* OP50 bacteria, with an OD600 of 1.6 or higher, were kept in the dark at 20 °C and used within a month.

### 2.3. Body Length

After exposure to B[a]P, the larvae were thoroughly washed with M9 buffer and then transferred to fresh NGM plates. M9 buffer was composed of 1 M MgSO_4_, 0.086 M NaCl, 0.022 M KH_2_PO_4_, and 0.017 M Na_2_HPO_4_·12H_2_O. Once the excess liquid evaporated, the worms migrated onto the bacterial lawn [30]. Fluorescent images of *C. elegans* were captured using a BX51 fluorescence microscope. Image analysis was performed with ImageJ software (version 1.54d) to measure the body length of the nematodes. For each concentration group, data were collected from 15 nematodes.

### 2.4. Brood Size

After exposure to B[a]P, the larvae were thoroughly washed with M9 buffer and then transferred to fresh NGM plates. The plates were replaced every 24 h, and the nematodes were monitored until they ceased laying eggs. The number of offspring for each group was observed and recorded, with data collected from 15 nematodes per concentration group.

### 2.5. Locomotor Behavioral Parameters

The locomotor behavioral parameters of *C. elegans* encompass head thrashing frequency, pharyngeal pumping rate, and body bending rate. The head thrashing frequency was determined by placing *C. elegans* on the NGM surface. After allowing the nematodes to move freely, the number of body swings from one side to the other within 30 s was counted under a stereomicroscope [31]. The pharyngeal pumping frequency was measured by placing *C. elegans* on the NGM surface. After allowing the nematodes to move freely, the frequency of pharyngeal pumping events within one minute was counted using a stereomicroscope [32]. The body bend frequency was measured by placing *C. elegans* on the NGM surface. After allowing the nematodes to move freely, the number of sinusoidal movements along the body’s long axis within one minute was recorded under a stereomicroscope [33]. All data were collected from 15 nematodes per concentration group.

### 2.6. Lifespan Assay

After exposure to B[a]P, the larvae were thoroughly washed with M9 buffer and then transferred to fresh NGM plates. Survival was assessed under a fluorescence microscope by observing the nematodes’ response to needle contact. A positive survival response is indicated by any observable movement upon contact with the needle [34]. Survival data were collected from 15 nematodes per concentration group. Statistical analyses and survival curves were generated using GraphPad Prism software (version 10.0).

### 2.7. ROS Detection

After exposure to B[a]P, the nematodes were incubated with 500 μL of 2′,7′-dichlorodihydrofluorescein (DCFH-DA, 1 µM) in the dark at room temperature for two hours. Following the incubation, the supernatant was removed, and the nematodes were washed three times with M9 buffer. Next, *C. elegans* strains were anesthetized by exposing them to a levamisole hydrochloride solution. Fluorescence imaging was performed using a laser scanning confocal microscope with an excitation wavelength of 488 nm and an emission filter set to 510 nm. The data were reported as relative fluorescence units, with normalization to the autofluorescence. Data were collected from 15 nematodes per concentration group, and ROS levels were quantified using ImageJ software (version 1.54d).

### 2.8. RNA Sequencing and Data Peocessing

After exposure to B[a]P, L1 larvae were collected, and total RNA was extracted and stored at −80 °C. The integrity and quantity of RNA were accurately assessed using the Agilent 2100 Bioanalyzer. RNA-seq libraries were then prepared according to the Illumina protocol. Briefly, poly(A)-selected total RNA was purified and fragmented. Using random hexamer primers, the first-strand cDNA was synthesized from the fragmented mRNA, followed by second-strand cDNA synthesis. The resulting double-stranded cDNA underwent end repair, phosphorylation, and A-tailing. Adapter ligation and PCR amplification were subsequently performed, generating libraries suitable for clustering on an Illumina flow cell. Sequencing was conducted on the Illumina HiSeq 2500 platform, producing tens of millions of clusters in parallel [35]. To ensure the quality and reliability of downstream data analysis, raw reads were filtered by removing reads containing adapter sequences, reads with ambiguous nucleotides (N), and low-quality reads (where bases with Qphred ≤ 20 accounted for more than 50% of the read length). Clean reads were then subjected to quality metrics, including Q20, Q30, and GC content calculations.

Read alignment to the *C. elegans* reference genome was performed using the featureCounts program (version 1.5.0-p3), and gene-level counts and RPKM values were computed. The RPKM values were quantile-normalized and log2-transformed before conducting unsupervised hierarchical clustering and principal component analysis (PCA). Differential expression analysis between comparison groups was performed using the DESeq2 package (version 1.20.0). Genes with a *p*-value < 0.05 and a fold change in normalized RPKM greater than 1.5 were considered significantly differentially expressed. Gene Ontology (GO) and Kyoto Encyclopedia of Genes and Genomes (KEGG) enrichment analyses of the differentially expressed genes were conducted using the clusterProfiler package (version 3.8.1).

### 2.9. RT-qPCR

After treatment with B[a]P, approximately 200 µL of *C. elegans* was transferred into 1.5 mL centrifuge tubes, and 450 µL of TRIzol reagent was quickly added. The samples were subjected to three freeze–thaw cycles, alternating between immersion in liquid nitrogen and incubation in a 37 °C water bath. Total RNA was subsequently extracted using the Direct-zol™ RNA MiniPrep kit. cDNA synthesis was performed utilizing the ChamQ Blue Universal SYBR qPCR Master Mix. For RT-qPCR, a 20 µL reaction volume was prepared, and amplification was carried out on an ABI Step OnePlus System. Primer sequences for RT-qPCR are listed in Supplementary Table S1. The amplification protocol included an initial denaturation at 95 °C for 5 min, followed by 40 cycles of denaturation at 95 °C for 15 s, annealing at 60 °C for 30 s, and an extension at 72 °C for 30 s. A final melting curve analysis was performed between 65 °C and 95 °C. Actin served as the internal control, and gene expression was quantified using the 2-∆∆Ct method [36,37]. We summarize the real-time PCR primer sequences for the target genes in Table S1.

### 2.10. Data Analysis

Data were visualized using GraphPad Prism 10. Statistical analyses were conducted to evaluate differences between groups. Prior to analysis, all datasets were tested for normality using the Shapiro–Wilk test. Where necessary, data were log-transformed to meet the assumptions of normal distribution. The fluorescence intensity of ROS and col-19::GFP was quantified using ImageJ 1.51 software. A one-way ANOVA followed by Tukey’s post hoc test was applied for comparisons among multiple groups, while independent sample t-tests were used for two-group comparisons. Lifespan data were analyzed using the log-rank (Mantel–Cox) test. Data are presented as mean ± standard deviation (SD), and differences were considered statistically significant at *p* < 0.05. All values were normalized relative to the corresponding control group.

## 3. Results

### 3.1. Effects of B[a]P on Physiological Parameters and Oxidative Stress in C. elegans

**Figure 1:**
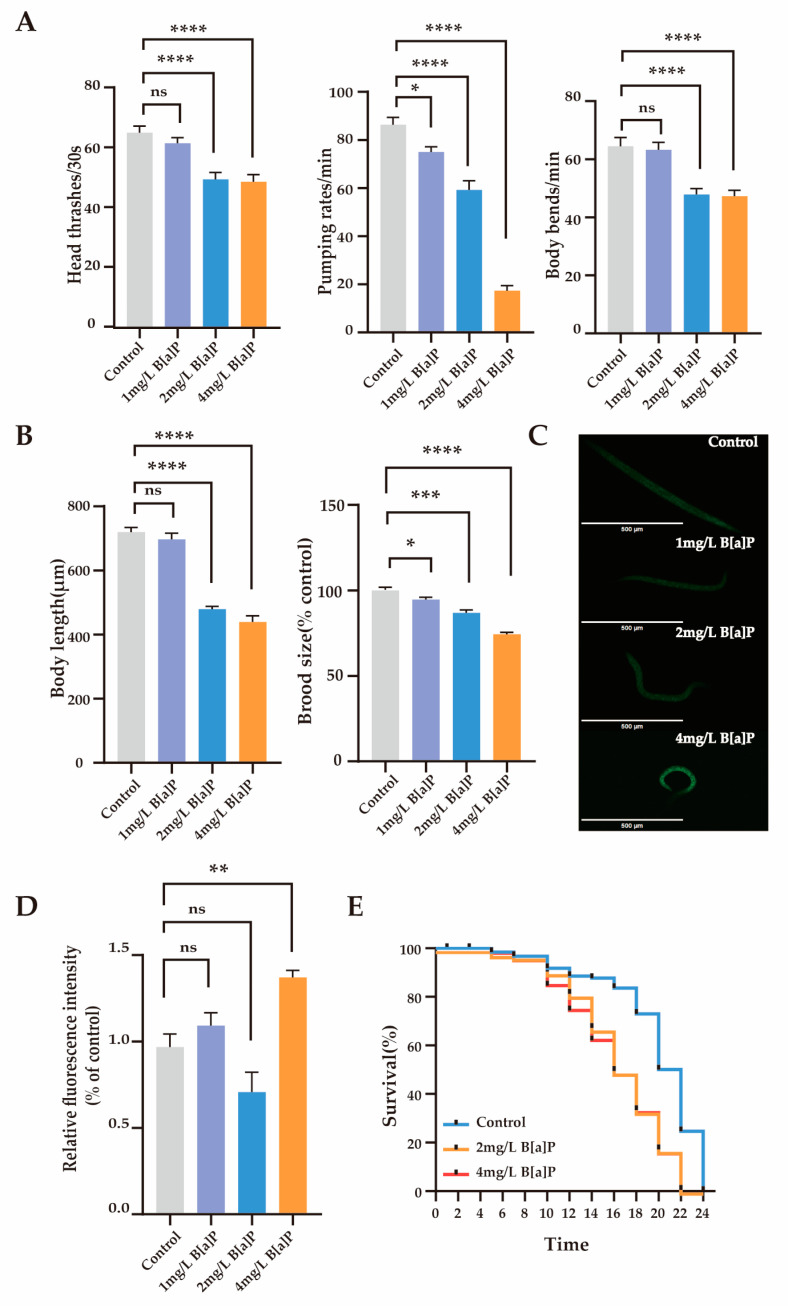
Effects of B[a]P toxicity on wild-type N2. (**A**) Head thrashing, pharyngeal pumping rate, and body bending; (**B**) body length and brood size; (**C**) representative fluorescent images of ROS; (**D**) quantification of ROS levels; (**E**) lifespan analysis. Data collected from 15 nematodes. * *p* < 0.05, ** *p* < 0.01, *** *p* < 0.001, **** *p* < 0.0001; ns denotes not significant.

To investigate the toxic effects of B[a]P, we measured the physiological indicators and oxidative stress expression levels in wild-type N2. We assessed the behavioral (head thrashing, pharyngeal pumping, and body bending frequency) and reproductive developmental indicators (body length and egg-laying rate) of *C. elegans*. The results showed that after 36 h of exposure to B[a]P, physiological parameters remained stable at a lower concentration (1 mg/L), whereas significant declines were observed at higher concentrations (2 to 4 mg/L) (*p* < 0.0001). As shown in Figure 1A, B[a]P at 2 and 4 mg/L significantly reduced head thrashing, pharyngeal pumping, and body bending frequency in wild-type N2. Moreover, both body length and egg-laying rate were significantly affected (*p* < 0.0001) (Figure 1B). Compared to the control group, higher concentrations of B[a]P (2 to 4 mg/L) markedly impaired locomotor function and reproductive development in wild-type N2, while the effects at 1 mg/L were not statistically significant.

Reactive oxygen species (ROS) are highly reactive oxygen molecules that reflect an organism’s response to environmental pollutants [38]. To assess the impact of B[a]P on oxidative stress, we measured the ROS levels in *C. elegans*. Compared to the control group, the high-dose groups showed a significant increase in ROS fluorescence expression levels, rising by 41% (Figure 1C,D). In contrast, ROS levels in the 1 mg/L and 2 mg/L treatment groups did not differ significantly from those in the control group. Overall, high concentrations of B[a]P substantially reduced physiological function in *C. elegans* and induced oxidative stress. Therefore, we focused on the toxic effects of B[a]P at higher concentrations (2 mg/L and 4 mg/L) to investigate its underlying molecular mechanisms. Figure 1E shows that the maximum lifespan of the control group, 2 mg/L B[a]P group, and 4 mg/L B[a]P group were 24 days, 22 days, and 22 days, respectively. Compared to the control group, the maximum lifespan of *C. elegans* in the B[a]P groups was reduced by 2 days. These findings indicate that B[a]P, at certain concentrations, adversely affects the survival of *C. elegans*.

### 3.2. Effect of B[a]P on Development of C. elegans

**Figure 2:**
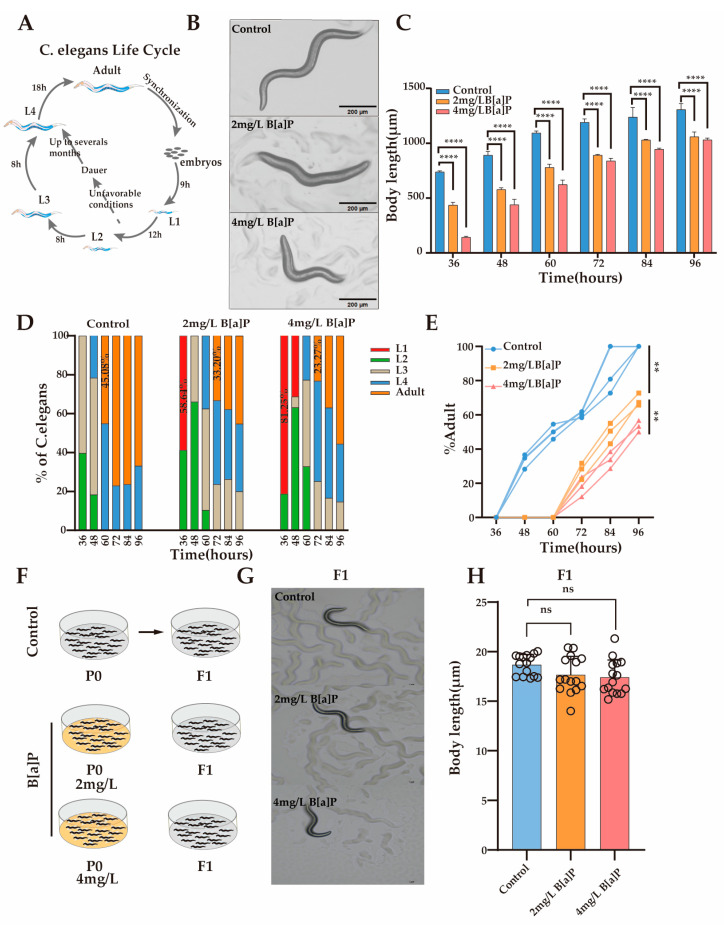
Effects of B[a]P on the development of wild-type N2 nematodes. (**A**) Diagram of the nematode developmental cycle; (**B**) representative images of parental nematodes; (**C**) body length measurements of parental nematodes; (**D**) proportion of nematodes at different developmental stages; (**E**) proportion of adults in the population; (**F**) schematic of B[a]P-exposed parents and normal culture of F1 offspring; (**G**) representative images of F1 offspring; (**H**) body length of F1 offspring. Data collected from 15 nematodes. ** *p* < 0.01, **** *p* < 0.0001; ns denotes not significant.

Body length is a crucial developmental indicator in *C. elegans*, with its development characterized by changes in body length and classified into distinct developmental stages. To investigate the impact of B[a]P exposure on nematode development, we monitored changes in body length under different concentrations of B[a]P. Under normal conditions, L1 larvae develop into adults within 46 h (Figure 2A). Our results showed that after 36 h of exposure, body length was significantly inhibited in a dose-dependent manner compared with the control group (Figure 2B). To further evaluate the developmental effects of B[a]P, we measured changes in body length every 12 h until egg laying. Although body length increased over time in all groups, worms exposed to B[a]P remained consistently shorter than those in the control group (Figure 2C).

To determine whether the inhibition of body length development was attributable to B[a]P exposure, we monitored the developmental progression of the nematodes under identical conditions (Figure 2D). At 36 h, worms in the control group developed to the L2/L3 stage, whereas 58.64% and 81.25% of the worms exposed to 2 mg/L and 4 mg/L B[a]P, respectively, remained at the L1 stage. At 60 h, 45.08% of the control group nematodes had developed into adults, while no adults were observed in the B[a]P-treated groups. At 72 h, 33.20% of worms in the 2 mg/L group and 23.27% in the 4 mg/L group developed into adults. These results indicate that B[a]P exposure delays developmental progression and reduces the number of mature adults by approximately half (Figure 2E). To assess the potential genotoxic effects of B[a]P, we cultured the F1 progeny of B[a]P-exposed worms under normal conditions and measured their body length (Figure 2F). There were no significant differences in body length between the F1 generation of the B[a]P-treated and control groups (Figure 2G,H). In summary, B[a]P exhibits pronounced developmental toxicity in *C. elegans*, as evidenced by reduced body length, a prolonged larval-to-adult transition time, and decreased adult yield.

### 3.3. Effects of B[a]P Exposure on Gene Expression in C. eleganss

**Figure 3:**
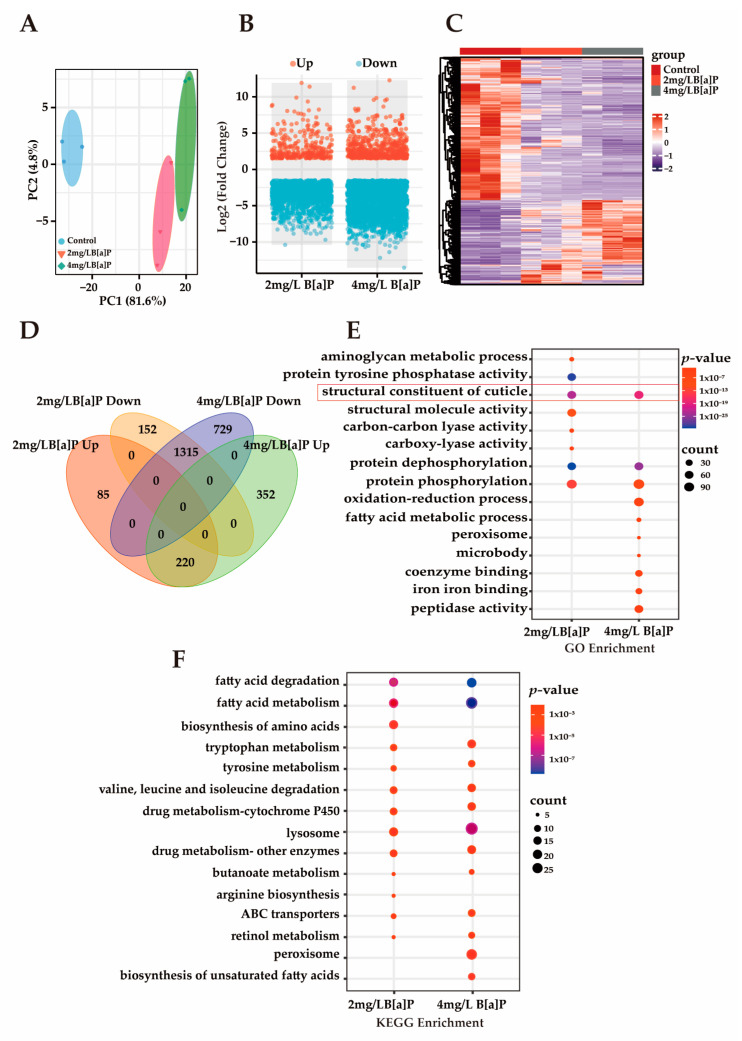
The transcriptomics analysis of *C. elegans*. (**A**) PCA; (**B**) volcano plot of DEGs; (**C**) heatmap of DEGs; (**D**) Venn diagram of DEGs across groups; (**E**) GO enrichment analysis of commonly downregulated DEGs; (**F**) KEGG enrichment analysis of commonly downregulated DEGs.

Multiple studies have demonstrated that B[a]P induces toxic effects during early developmental stages in organisms. However, its underlying molecular mechanisms remain unclear [39,40]. Our preliminary experiments showed that B[a]P exerts significant developmental toxicity in wild-type N2 *C. elegans*, as evidenced by markedly shortened body length, delayed progression from larva to adult, and a reduced proportion of adult worms. To investigate B[a]P-induced gene expression changes and explore its potential molecular mechanisms of developmental toxicity, we conducted RNA sequencing (RNA-seq). As shown in Figure 3A, principal component analysis (PCA) revealed a clear separation between the treatment and control groups, with tight clustering within each group, indicating high sample representativeness and experimental reliability. Differentially expressed genes (DEGs) were identified based on a threshold of *p*-value < 0.05 and an absolute fold change in normalized RPKM greater than 1.5. Volcano and heatmap visualizations of these DEGs revealed that most DEGs were downregulated (Figure 3B,C). Among the DEGs consistently altered in both the 2 mg/L and 4 mg/L B[a]P treatment groups, 1315 genes were downregulated and 220 genes were upregulated (Figure 3D). The predominant downregulation of gene expression may be associated with B[a]P-induced developmental delay. Enrichment analysis of these downregulated DEGs was performed to identify affected pathways. GO analysis revealed a significant enrichment of the “structural constituent of cuticle” term, suggesting impaired cuticle formation (Figure 3E). Additionally, genes involved in protein phosphorylation and dephosphorylation were significantly downregulated, indicating that B[a]P exposure suppresses protein metabolism in *C. elegans*. KEGG pathway analysis further demonstrated that B[a]P downregulated multiple metabolic pathways, including those related to amino acid metabolism, fatty acid metabolism, and lysosomal function, with the lysosome pathway being significantly enriched (Figure 3F). Lipl-1 is a lysosomal lipase gene, and B[a]P significantly reduced lipl-1 expression (Figure S1). A developmental study on *C. elegans* larvae uncovered a signaling pathway that transduces signals from the ECM to the cell nucleus, activating lysosomes to promote the extensive ECM remodeling that is crucial for larval development [41]. This extensive ECM remodeling presents a significant challenge to the underlying epidermis responsible for the cuticle. The dynamic regulation of lysosomes plays a critical role in cellular and tissue homeostasis by molecularly regulating ECM synthesis and mediating the developmental process of *C. elegans* [42]. Therefore, lysosomes are essential for ECM remodeling during *C. elegans* development, and the inhibition of lysosomal activity disrupts cuticle shedding and leads to collagen deposition disorders.

### 3.4. Effects of B[a]P on Cuticle Collagen Gene Expression in C. elegans

**Figure 4:**
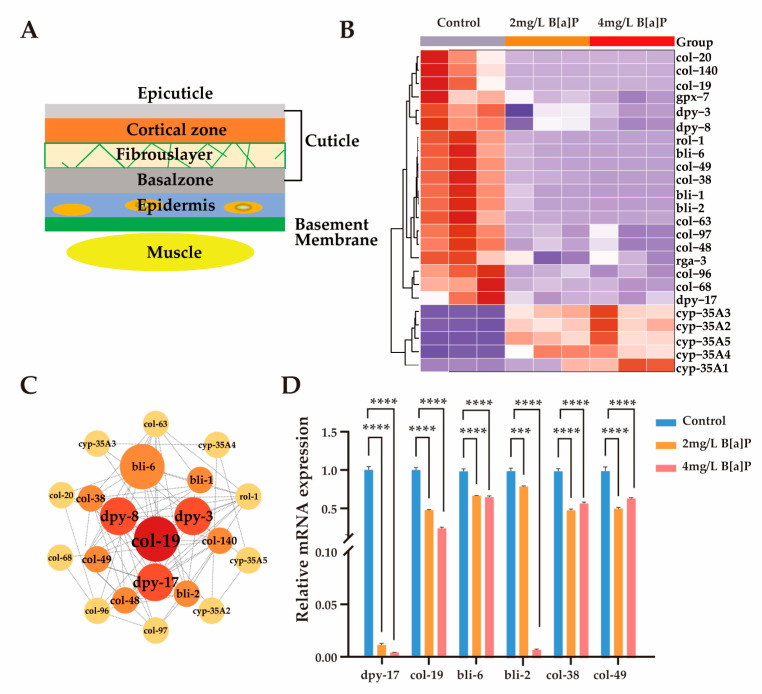
Effect of B[a]P on the expression of cuticle-related genes. (**A**) Schematic of cuticle structure; (**B**) heatmap of differentially expressed cuticle-related genes; (**C**) interaction network of cuticle collagen genes; (**D**) mRNA levels of six key cuticle synthesis genes; *** *p* < 0.001, **** *p* < 0.0001.

The mechanisms by which *C. elegans* perceives its body size and initiates molting are partially regulated by the structural properties of its collagen-rich cuticle [41,43,44]. Mutations in genes encoding cuticular collagens frequently result in morphological abnormalities in *C. elegans* [41,45,46]. As illustrated in Figure 4A, the cuticle is synthesized and secreted by the underlying hypodermis, which is subdivided into basal, medial, cortical, and apical regions [47]. Composed primarily of cross-linked collagens, insoluble keratins, glycoproteins, and lipids, the cuticle serves as a crucial protective barrier against environmental stressors during development [45].

To investigate whether the developmental delay induced by B[a]P exposure is associated with disruptions in collagen biosynthesis, we analyzed the expression of genes involved in cuticle formation. Given space constraints, we present only a subset of representative collagen biosynthetic genes and members of the cyp-35A gene family. Exposure to B[a]P led to a marked upregulation of cyp-35A gene expression, accompanied by a notable downregulation of collagen biosynthesis genes (Figure 4B). These results align with previous reports indicating that the cyp-35 pathway is involved in B[a]P metabolism [28,48]. Subsequent gene interaction network analysis identified col-19 and members of the dpy gene family as central components of the cuticular collagen gene cluster (Figure 4C). To validate the transcriptomic findings, we quantified the mRNA levels of key collagen biosynthesis genes (dpy-17, col-19, bli-2, bli-6, col-38, and col-49), as well as cyp-35A family members (cyp-35A2, cyp-35A5). In agreement with RNA sequencing results, the expression of collagen biosynthetic genes was significantly reduced, while cyp-35A gene expression was substantially elevated (Figure 4D and Figure S1). Collectively, these findings suggest that B[a]P exposure significantly suppresses the expression of genes involved in lysosomal function and cuticular collagen synthesis.

### 3.5. Molecular Mechanisms Underlying Developmental Toxicity of B[a]P

**Figure 5:**
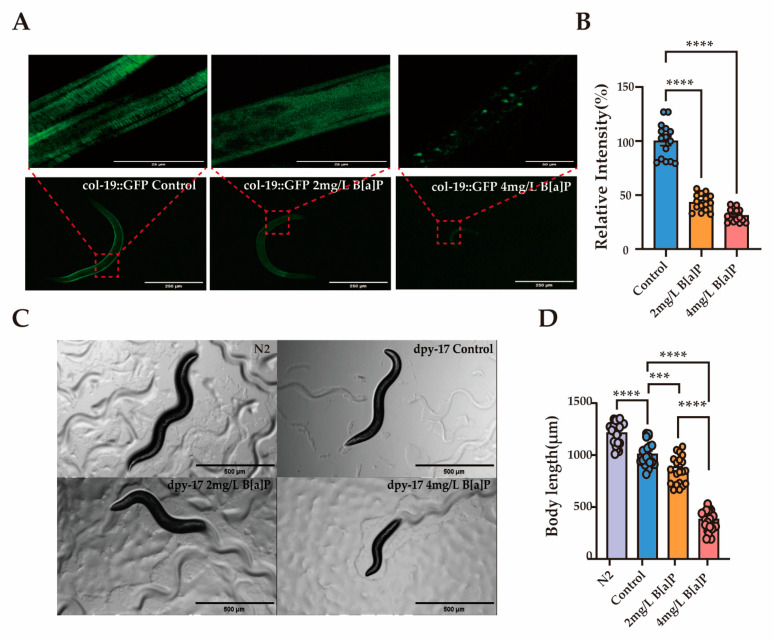
Effects of B[a]P on col-19::GFP and dpy-17 mutant *C. elegans*. (**A**) Representative fluorescence images of col-19::GFP nematodes; (**B**) quantification of fluorescence intensity in col-19::GFP nematodes; (**C**) representative images of dpy-17 mutant nematodes; (**D**) body length measurements of dpy-17 mutants; data collected from 15 nematodes. *** *p* < 0.001, **** *p* < 0.0001.

Col-19 is an adult-specific cuticle protein and serves as a critical marker for identifying defects in the cuticle ECM morphology in adult *C. elegans* [49]. The dpy-17 gene encodes a collagen protein that is essential for maintaining the structure and development of the skin and epidermis [50]. To explore the link between collagen and B[a]P-induced developmental delay, we used transgenic worms expressing GFP-tagged col-19 and dpy-17 mutants. Exposure to B[a]P significantly reduced the average fluorescence intensity in col-19::GFP transgenic worms. Moreover, the cuticle structure of these worms was visibly disrupted. The normally clear and regularly spaced striations were replaced by a diffuse and blurred fluorescence pattern (Figure 5A,B). In dpy-17 mutant worms, body length decreased progressively with increasing B[a]P concentrations (Figure 5C,D).

**Figure 6:**
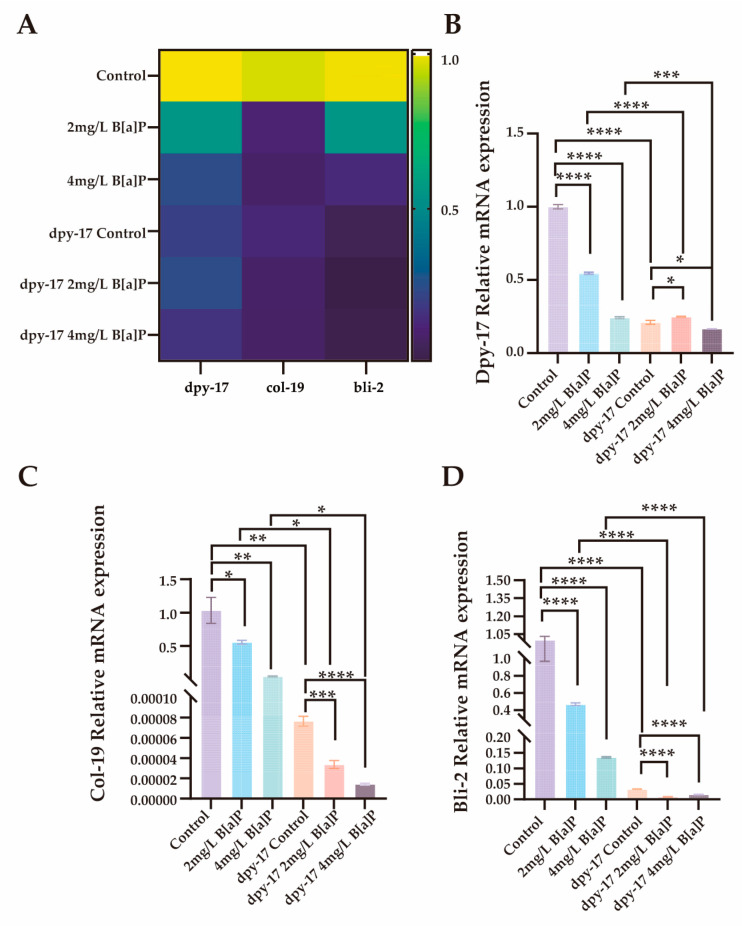
Effects of B[a]P on the expression of dpy-17, col-19 and bli-2 in *C. elegans*. (**A**) Heatmap of dpy-17, col-19, and bli-2 expression; (**B**) MRNA levels of dpy-17 in N2 and dpy-17 mutant nematodes; (**C**) MRNA levels of col-19 in N2 and dpy-17 mutant nematodes; (**D**) MRNA levels of bli-2 in N2 and dpy-17 mutant nematodes; * *p* < 0.05, ** *p* < 0.01, *** *p* < 0.001, **** *p* < 0.0001.

In *C. elegans*, the significant downregulation of dpy-17 may impair collagen synthesis and disrupt the structural integrity of the exoskeleton [51]. In addition to dpy-17 and col-19, the cuticle collagen gene bli-2 (nematode cuticle collagen N-terminal domain-containing protein) also plays a critical role in cuticle synthesis [50,52,53]. To evaluate the effects of B[a]P exposure on collagen synthesis, we performed RT-qPCR analysis on wild-type N2 and dpy-17 mutant worms after 36 h of B[a]P treatment. We quantified the expression of three key genes involved in cuticle collagen synthesis, namely dpy-17, col-19, and bli-2. As shown in Figure 6, all three genes exhibited significantly reduced expression in a concentration-dependent manner compared to the control group. These results not only confirm dpy-17 as a valid mutant model for impaired collagen production but also suggest that col-19 and bli-2 may act downstream of dpy-17 and contribute to cuticle collagen biosynthesis. Collectively, our findings indicate that the developmental toxicity induced by exogenous B[a]P exposure, as evidenced by reduced body length, is closely associated with impaired collagen synthesis.

Previous studies have demonstrated that the IIS signaling pathway plays a critical role in the development of *C. elegans*. DAF-2, a homolog of the insulin-like growth factor 1 receptor, regulates the nuclear localization and activity of the FOXO transcription factor daf-16 by activating downstream kinases such as age-1 (PI3K) and akt-2 [54]. When the DAF-2 function is altered, DAF-16 translocates to the nucleus, where it governs the expression of genes involved in longevity, stress resistance, and development [55]. To investigate whether B[a]P modulates collagen gene expression through the IIS pathway, we conducted RT-qPCR analysis of key transcription factors within this signaling cascade. Following B[a]P exposure, *daf-2* expression was upregulated, along with its downstream effectors *age-1* and *akt-2*. This activation led to the suppression of *daf-16*, evidenced by its reduced expression (Figure S2). These findings suggest that the IIS pathway may contribute to the developmental effects observed following B[a]P exposure.

## 4. Discussion

B[a]P is a well-established carcinogen extensively studied in various model organisms. Human exposure to B[a]P primarily occurs through cigarette smoking and dietary intake. Research has confirmed that B[a]P in cigarette smoke significantly increases the risk of developing lung cancer [56,57]. For non-smokers, diet represents the main route of B[a]P exposure. Numerous animal studies have demonstrated the developmental toxicity of B[a]P. Maternal dietary intake of B[a]P during pregnancy has been associated with reduced birth weight in offspring [13]. In dechorionated zebrafish embryos, exposure to 0.5, 5, and 50 nM B[a]P for 48 h post-fertilization (hpf) induced AHR-dependent cerebrovascular defects [58]. In rats, exposure to environmentally relevant doses of B[a]P (0, 0.1, 1.0, or 10 μg/kg) during organogenesis adversely affected fetal development [14]. Another rat study reported that maternal B[a]P exposure during pregnancy disrupted the hippocampal synaptic ultrastructure in offspring, leading to neurobehavioral impairments [59]. Additionally, B[a]P exposure significantly affected black carp larvae, resulting in reduced hatching rates, increased malformation rates, and growth delay [40]. However, due to ethical constraints and the long lifespan of traditional animal models, most studies on the developmental toxicity of B[a]P have focused on specific developmental stages, making it challenging to comprehensively evaluate the impacts of B[a]P across different life stages and on the entire organism. To date, the mechanisms underlying B[a]P-induced developmental toxicity remain unclear. *C. elegans*, with its short lifespan and clearly defined developmental stages, provides an excellent model for developmental toxicology studies.

We employed the *C. elegans* model to investigate the effects of B[a]P on early-life stage development. A range of physiological indicators, including locomotor behavior, body length, and brood size, are commonly used to evaluate the toxic effects of environmental pollutants on *C. elegans* [60,61]. Our results showed that B[a]P (2 and 4 mg/L) significantly impaired locomotor behavior and reproductive development, indicating its developmental toxicity in *C. elegans*. Jorge et al. [62] revealed that exposure to B[a]P, both before and after puberty, negatively impacts the reproductive health of the F1 offspring in rats. Further studies revealed that 0.1 μg/kg of B[a]P impaired sexual development and reproductive capacity in the F2 generation of rats [63]. Unusually, our study did not observe differences in the body length of F1 generation *C. elegans*. This could be attributed to the fact that the developmental toxicity of B[a]P in the parental generation (F0) did not lead to marked alterations in gene expression in the subsequent generation. Additionally, in the absence of B[a]P exposure, the presence of food might promote partial recovery in *C. elegans*, enabling the resumption of normal egg-laying and reproductive activities. Food provides essential energy for growth, development, and tissue repair [64], which may explain the partial recovery from B[a]P-induced developmental impairments. In summary, our findings indicate that B[a]P significantly reduces the number of *C. elegans* larvae reaching adulthood and delays the larval-to-adult developmental transition without causing transgenerational effects.

Col-19 is a cuticle protein specifically expressed in adult *C*. *elegans*, serving as a key marker for ECM abnormalities in the adult cuticle [49]. Using transgenic worms expressing green fluorescent protein-tagged col-19, we observed that exposure to B[a]P significantly reduced col-19 fluorescence and disrupted cuticle integrity. Furthermore, using RNAi-deficient mutant worms, we found that B[a]P exacerbated the dwarf phenotype of dpy-17 mutants. These findings suggest that B[a]P impairs cuticle biosynthesis, thereby hindering developmental progression. Previous studies have shown that dpy-17 promotes cuticle synthesis and assembly during early secretion processes [50]. Further study demonstrated that the genes dpy-17 and col-121 have a direct influence on the regulation of the collagenous cuticle, which is crucial for proper *C. elegans* development and reproduction [53]. Disruptions in these genes’ functions can cause defects in cuticle formation, resulting in abnormal organ development, morphological irregularities, impaired growth, delayed embryonic development, and defects in muscle formation [45]. Collectively, these studies support the notion that the disruption of the cuticle structure delays larval development and impedes the normal transition from larva to adult in *C. elegans*.

In this study, we identified a developmental toxic effect of B[a]P and sought to elucidate its underlying molecular mechanisms. B[a]P is metabolized in *C. elegans* primarily via the cyp-35A pathway, which induces xenobiotic stress responses. The IIS pathway plays a central role in regulating developmental processes. In our experiments, B[a]P exposure upregulated cyp-35A expression while downregulating genes involved in lysosomal lipolysis and cuticular collagen biosynthesis. Moreover, B[a]P activated the IIS pathway by stimulating the daf-2 receptor, leading to increased expression of downstream kinases such as age-1 and akt-2 and subsequent inhibition of the FOXO transcription factor daf-16. This signaling pattern may be associated with impaired lysosomal function and reduced expression of ECM-related genes, potentially compromising ECM remodeling. As a result, B[a]P-exposed worms appear unable to mount effective adaptive responses to environmental stress, thereby experiencing disrupted development. Although our data suggest a potential link between early B[a]P exposure and developmental abnormalities in *C. elegans*, we cannot rule out the involvement of additional pathways or contributing factors. Further studies are warranted to elucidate these mechanisms in greater detail. Overall, our findings highlight the need for heightened awareness and precautionary measures during critical periods of early development, including minimizing dietary intake of B[a]P-containing foods such as grilled or charred items.

## 5. Conclusions

Exposure to corresponding concentrations of B[a]P induces developmental toxicity in *C*. *elegans*, characterized by reduced body length, delayed maturation, and decreased maturation rate. Our study uncovers the potential molecular mechanism underlying the developmental toxicity of B[a]P. B[a]P may impair collagen synthesis during the development of *C. elegans* by modulating the daf-2/daf-16 IIS pathway. This reduction in collagen production likely compromises the effective remodeling of the ECM, weakening the organism’s ability to withstand environmental stress and ultimately resulting in developmental delay. These findings provide important insights for further investigations into the developmental toxicity mechanisms of B[a]P and contribute to future health risk assessments.

## Data Availability

The original contributions presented in this study are included in the article; further inquiries can be directed to the corresponding authors.

## Supplementary Materials

The following supporting information can be downloaded at: https://www.mdpi.com/article/10.3390/toxics13050384/s1, Figure S1: The mRNA expression levels of lipl-1, cyp-35A5 and cyp-35A2; Figure S2: The mRNA expression levels of key transcription factors in the IIS pathway; Table S1: RT-qPCR Primer Sequences; Excel: Raw data of the transcriptome in Figure 3.
