# Supplementary material for: Benzo[a]pyrene-Induced Developmental Toxicity in Caenorhabditis elegans: Potential Involvement of Insulin/IGF Signaling and Collagen Gene Dysregulation
Source: Toxics. 2025 May 9;13(5):384. doi: 10.3390/toxics13050384 (PMC12115400; doi:10.3390/toxics13050384)
Supplement: Supplementary file 1 [file toxics-13-00384-s001.zip › Supplementary material.pdf]

Supplementary Data

Benzo[a]pyrene-Induced Developmental Toxicity in Caenorhabditis elegans: Potential Involvement of Insulin/IGF Signaling and Collagen Gene Dysregulation

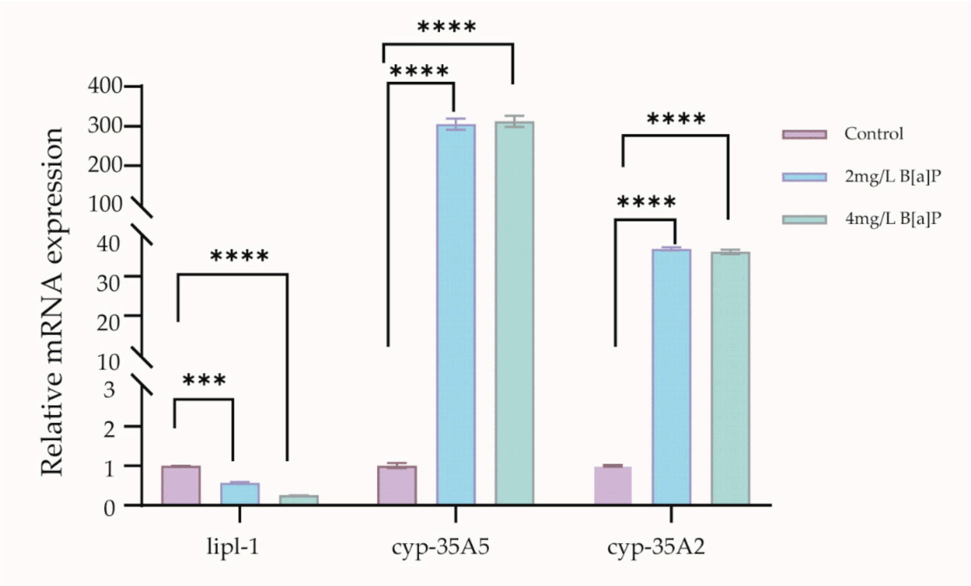

Supplemental FigureS1 (Related to figure 4D). The mRNA expression levels of lipl-1, cyp-35A5 and cyp-35A2. Statistical significance was determined by comparing differences with the control, where p-values of ***< 0.001, ****< 0.0001.

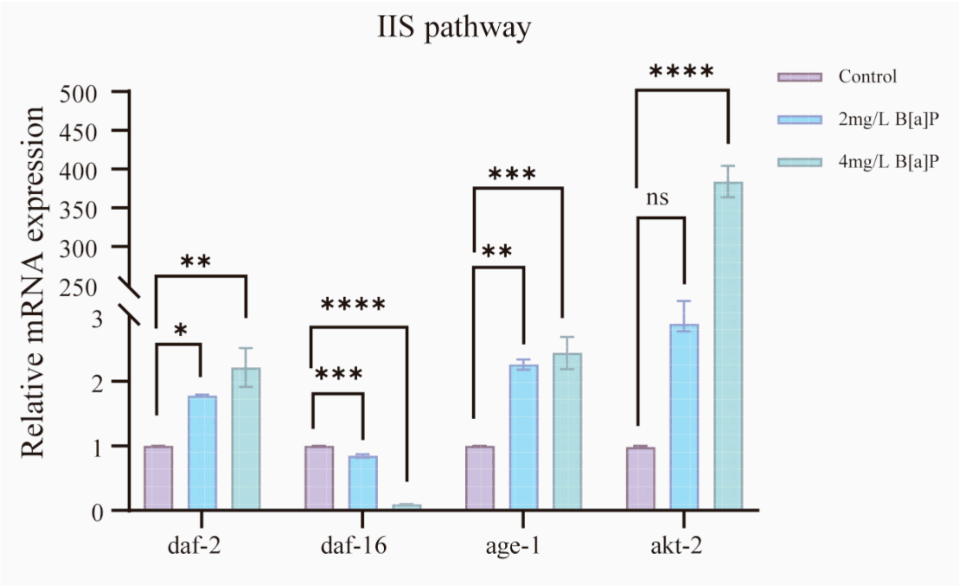

Supplemental FigureS2 (Related to Graphical Abstract). The mRNA expression levels of key transcription factors in the IIS pathway. Statistical significance was determined by comparing differences with the control, where p-values of *< 0.05, **< 0.01, ***< 0.001, ****< 0.0001, and ns (not significant).

Supplemental TableS1. RT-qPCR Primer Sequences.

Gene name	Species	Sequence (from 5′ to 3′)
Col-19-F	C. elegans	TAGAGCTATCACCCAAGACA
Col-19-R	C. elegans	GCAGTTACATTGCTCGAATC
Dpy-17-F	C. elegans	CCACAAGTCTATCAGCAAGT
Dpy-17-R	C. elegans	TGTAGATTTGATCGGACTCG
Bli-2-F	C. elegans	GGTAGAGATGGAAAACCAGG
Bli-2-R	C. elegans	CTACAGGGCAATCGTAACAT
Bli-6-F	C. elegans	GAAGGGAATCTGTCCAAAGT
Bli-6-R	C. elegans	ATCAACAGTTTATCGTCGGG
Col-38-F	C. elegans	TGGAGTATCTCTGGCTGTTA
Col-38-R	C. elegans	GTCCTTGTTCTCCAGTGATT
Col-49-F	C. elegans	CCAGGAGTTGATGGAAAAGA
Col-49-R	C. elegans	CGAATCAAGCAGTTCTAGGT
Daf-2-F	C. elegans	GTTGATAATGCTGCCGAG
Daf-2-R	C. elegans	ATCCCGGTCCGATTTCTT
Daf-16-F	C. elegans	TCGTCGTCTCGTGTTTCTCCA
Daf-16-R	C. elegans	TTCCATAGGCACCCGGTAGTG
Age-1-F	C. elegans	AGCACACCAAAACAGAGCAAGA
Age-1-R	C. elegans	CGTCAAGGTCCCAAAGTGAAA
Akt-2-F	C. elegans	AAGCCCAGAAGCCGTAACAT
Akt-2-R	C. elegans	GAATTGAAACGTAACGTACTCTGTC
Act-1-F	C. elegans	TCTCCAAGCAAGAATACGAC
Act-1-R	C. elegans	AATAGAAAGCTGGTGGTGAC

Col-19,Cuticle collagen 19; Dpy-17, Cuticle collagen dpy-17; Bli-2, Nematode cuticle collagen N-terminal domain-containing protein; Bli-6, Nematode cuticle collagen N-terminal domain-containing protein; Col-38, Cuticle collagen 38; Col-49, Cuticle collagen 49; Daf-2, Insulin-like receptor subunit beta; Protein kinase domain-containing protein; receptor protein-tyrosine kinase; Daf-16, Forkhead box protein O; Age-1, Phosphatidylinositol 3-kinase age-1; Akt-2, Serine/threonine-protein kinase akt-2; Act-1, Actin-1.
